# The efficacy and safety of cyclosporine in children with systemic lupus erythematosus: A protocol for systematic review and meta-analysis

**DOI:** 10.1097/MD.0000000000032314

**Published:** 2023-02-22

**Authors:** Xiaohui Liu, Yu Peng, Sufen Cai

**Affiliations:** a Department of Rheumatology and Immunology, Jiangxi Provincial Children’s Hospital, Jiangxi, China.

**Keywords:** cyclosporine, efficacy, meta-analysis, systemic lupus erythematosus

## Abstract

**Background:**

Childhood-onset systemic lupus erythematosus (SLE) is a rare but severe multisystem autoimmune/inflammatory disease with marked heterogeneity between patients, causing anything from mild to life-threatening disease. We performed a protocol for systematic review and meta-analysis to evaluate the efficacy and safety of cyclosporine in childhood-onset SLE.

**Methods:**

This systematic review has been registered in PROSPERO (CRD42022377450), which will be conducted in accordance with Preferred Reporting Items for Systematic Review and Meta-Analysis Protocols 2015 statement. Only randomized controlled trials will be included.

We searched the following databases including PubMed, EMBASE, the Cochrane Library, SinoMed, CNKI, VIP, Wanfang Data and International Clinical Trials Register Search Portal, and Clinical Trials.gov. Two researchers will use the Cochrane systematic evaluation tool to assess the risk of bias independently. Data synthesis will be performed using RevMan V.5.4.

**Results:**

This study will comprehensively summarize the high-quality trials to determine the efficacy and safety of cyclosporine in the treatment of childhood-onset SLE.

**Conclusion:**

This study may be beneficial to health policymakers, clinicians, and patients with regard to the use of cyclosporine in childhood-onset SLE.

## 1. Introduction

Systemic lupus erythematosus (SLE) has a wide range of clinical and laboratory findings and has variable prognoses, depending upon the severity and type of organ involvement.^[1–3]^ Clinical course and outcome can no longer be considered separately from the results of treatment. Although recent advances in diagnosis and treatment have led to a remarkable improvement in the prognosis of SLE, 5 to 20% of patients continue to progress to end-stage renal disease within 10 years following the diagnosis of nephritis.^[4,5]^

Childhood-onset SLE is rare, with an estimated annual incidence of 0.3 to 0.9/100,000 children.^[6,7]^ The heterogeneous and complex nature of SLE means that diagnosis is often delayed until progression is advanced and several organs have become affected. This is particularly pronounced in childhood-onset SLE, in which children frequently experience higher disease activity and a faster rate of SLE damage accrual than adults.^[8]^ Several studies have compared the potential differences between children and adults with SLE; children used to have more active disease at presentation and over time than do adults with SLE and they have often an aggressive clinical course and more frequent renal involvement as compared to adults; therefore, children probably receive more intensive drug therapy and accrue more damage.^[9,10]^

Pediatric patients are typically treated with combinations of corticosteroids, immunosuppressants, antimalarials, and non-steroidal anti-inflammatory drugs, although none are approved. Recent trials have suggested that calcineurin inhibitors including cyclosporine may be a potential option for SLE.^[11]^ Cyclosporine binds to cyclophilin and inhibits calcineurin, thus inhibiting the production and release of interleukin-2 and subsequent interleukin-2-induced activation of resting T-lymphocytes.^[12]^ Cyclosporine can also reduce proteinuria through the stabilization of the actin cytoskeleton in renal podocytes. However, few studies have reported the clinical use of cyclosporine for treating childhood-onset SLE. Thus, we performed a protocol for systematic review and meta-analysis to evaluate the efficacy and safety of cyclosporine in childhood-onset SLE.

## 2. Methods

### 2.1. Study registration

This protocol has been registered on PROSPERO (registration number: CRD42022377450). We will report it according to the Preferred Reporting Items for Systematic Reviews and Meta-Analysis Protocols guidelines and meta-analysis protocols.^[13]^ Ethics approval is not required for systematic review. Additionally, peer-reviewed publications will publish the current review’s findings.

### 2.2. Eligibility criteria

#### 2.2.1. Population.

All the patients enrolled in the study are children (<18 years old) and are diagnosed with SLE. There will be no restrictions on gender, race, nationality, the severity of disease, or the duration of treatment of the participants.

#### 2.2.2. Interventions and comparisons.

Patients in the intervention groups receive cyclosporine combined with glucocorticoid. The comparison groups receive glucocorticoids alone. There will be no restrictions on the type of glucocorticoids, dose of cyclosporine, or course of treatment.

#### 2.2.3. Outcomes.

The primary outcomes are the clinical symptoms, SLE disease activity index, and quality of life questionnaire. The secondary outcomes are C3 levels, hemoglobin levels, white blood cell levels, and adverse reactions.

#### 2.2.4. Design.

Only randomized controlled trials (RCTs) will be included in this meta-analysis.

### 2.3. Exclusion criteria

The following studies will be excluded: repeated publications, review of literature and cases, animal studies, incomplete literature, and non-RCTs or RCTs involving adult patients.

### 2.4. Search methods

We searched the following databases including PubMed, EMBASE, the Cochrane Library, SinoMed, CNKI, VIP, Wanfang Data, and International Clinical Trials Register Search Portal and Clinical Trials.gov to identify RCTs involving the above-mentioned interventions. We will conduct the literature search from the inception of all the databases to November 2022 and will update it before the review is completed. Studies in accordance with the PICOS will be considered. Key search terms (MeSH and Free words) used for our searches are “systemic lupus erythematosus,” “cyclosporine,” “children” and “RCTs.” The search strategy in PubMed is shown in Table 1.

**Table 1:** Search strategy for the PubMed database.

#1 systemic lupus erythematosus [Title/Abstract]
#2 SLE [Title/Abstract]
#3 lupus kidney [Title/Abstract]
#4 #1 OR #2 OR #3
#5 child [Title/Abstract]
#6 children [Title/Abstract]
#7 juvenile [Title/Abstract]
#8 young [Title/Abstract]
#9 adolescent [Title/Abstract]
#10 teenager [Title/Abstract]
#11 #5 OR #6 OR #7 OR#8 OR#9 OR#10
#12 cyclosporine [Title/Abstract]
#13 cyclosporin [Title/Abstract]
#14 CsA [Title/Abstract]
#15 calcineurin inhibitors [Title/Abstract]
#16 #12 OR #13 OR #14 OR #15
#17 randomized controlled trial[Publication Type]
#18 randomized [Title/Abstract]
#19 randomly [Title/Abstract]
#20 #17 OR #18 OR #19
#21 #4 AND #11 AND #16 AND #20

### 2.5. Study selection

**Figure 1.**
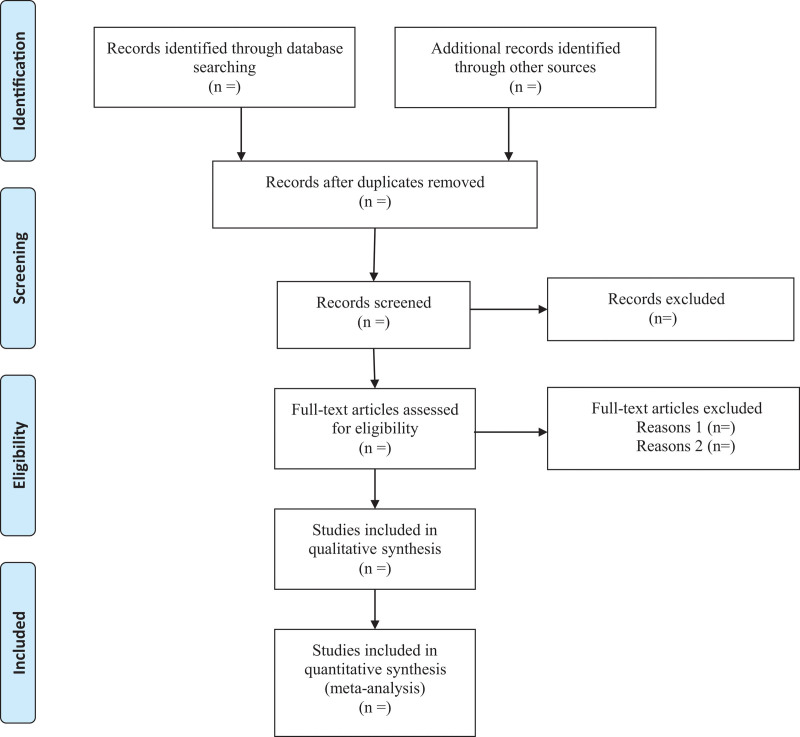
PRISMA flow chart of the study selection process.

All retrieved records will be imported into Endnote X9.1 software and the duplicated records will be removed. For studies that have been updated, the older ones will be excluded or can be used as supplementary data in further research. Titles and abstracts will be screened independently by 2 reviewers. Full texts will be obtained for eligible studies and will be screened independently. Discrepancies will be resolved through discussion, or by consulting a third reviewer. The procedures of study selection will be performed in accordance with the Preferred Reporting Items for Systematic reviews and Meta-Analysis flow charts (as shown in Fig. 1).

### 2.6. Data extraction

Two investigators will independently extract data from the selected articles. The extracted data will mainly include the first author, title, country, region, year of publication, sample size, study methods, interventions, patient characteristics, outcomes, and adverse events. Two investigators will enter the extracted data into Microsoft Excel and cross-check them. If there is any disagreement between the 2 reviewers during the screening process, it will be resolved by referring to the original article and discussing it with the third investigator.

### 2.7. Risk of bias

Two researchers will use the Cochrane systematic evaluation tool to assess the risk of bias in included trials independently.^[14]^ The bias tool embodies 7 components: random sequence generation, allocation concealment, blindness of participants and caregivers, blindness of outcome evaluators, incomplete outcome data, selective outcome reports, and other biases. Each item will be assessed on 3 levels: high risk, low risk, or unclear risk. All disagreements will be resolved by discussion with a third investigator to reach a consensus.

### 2.8. Data synthesis

Data synthesis will be performed using RevMan V.5.4 (Cochrane Collaboration). Results are expressed as risk ratios and standardized or weighted mean differences for continuous data. The methodology is as follows: if the *I*^2^ test is <50%, a fixed-effects model is used for data synthesis. If the *I*^2^ test is between 50 and 75%, a random-effects model is used for data synthesis. If the *I*^2^ test is >75%, we investigate possible causes from a clinical and methodological point of view and perform subgroup analysis. If the data cannot be synthesized, we provide descriptive analysis to address this issue. In cases where more than 10 trials meet the trial criteria, we will use the Revman 5.4 software to produce funnel plots to assess bias.

### 2.9. Evidence level

The evidence grade will be assessed using the guidelines of the Grading of Recommendations, Assessment, Development, and Evaluation working group including the following items: risk of bias, inconsistency, indirectness, imprecision, and publication bias.^[15]^

## 3. Discussion

Childhood-onset SLE is a rare but severe multisystem autoimmune/inflammatory disease with disease onset before the 18th birthday.^[16]^ It is a highly complex disease with marked heterogeneity between patients, causing anything from mild to life-threatening disease, following a relapsing and remitting course, and with an unpredictable natural history.

Treatment of childhood-onset SLE is complex and less standardized compared to adult-onset SLE.^[17]^ Regardless of differences in the pathophysiology, classification criteria, and commonly used treatment options are identical, and age-specific differences fail to be appreciated. Another layer of complexity is added in childhood-onset SLE with the occurrence of potentially toxic events and treatment-related side effects alongside the ongoing physical, mental and psychosocial developmental processes of childhood.^[18,19]^ This study provide evidence for the efficacy and safety of cyclosporine in childhood-onset SLE. To improve the prognosis, multi-disciplinary teams should be involved with both the initial diagnosis and subsequent management of disease flares and/or complications.

## Author contributions

**Conceptualization:** Yu Peng.

**Data curation:** Yu Peng.

**Writing – original draft:** Sufen Cai.

**Writing – review & editing:** Xiaohui Liu.
